# Cell volume regulation in epithelial physiology and cancer

**DOI:** 10.3389/fphys.2013.00233

**Published:** 2013-08-30

**Authors:** Stine F. Pedersen, Else K. Hoffmann, Ivana Novak

**Affiliations:** Department of Biology, University of CopenhagenCopenhagen, Denmark

**Keywords:** K^+^ channels, Cl^−^ channels, tumour microenvironment, drug resistance, pancreatic cancer, breast cancer, stroma, secretion

## Abstract

The physiological function of epithelia is transport of ions, nutrients, and fluid either in secretory or absorptive direction. All of these processes are closely related to cell volume changes, which are thus an integrated part of epithelial function. Transepithelial transport and cell volume regulation both rely on the spatially and temporally coordinated function of ion channels and transporters. In healthy epithelia, specific ion channels/transporters localize to the luminal and basolateral membranes, contributing to functional epithelial polarity. In pathophysiological processes such as cancer, transepithelial and cell volume regulatory ion transport are dys-regulated. Furthermore, epithelial architecture and coordinated ion transport function are lost, cell survival/death balance is altered, and new interactions with the stroma arise, all contributing to drug resistance. Since altered expression of ion transporters and channels is now recognized as one of the hallmarks of cancer, it is timely to consider this especially for epithelia. Epithelial cells are highly proliferative and epithelial cancers, carcinomas, account for about 90% of all cancers. In this review we will focus on ion transporters and channels with key physiological functions in epithelia and known roles in the development of cancer in these tissues. Their roles in cell survival, cell cycle progression, and development of drug resistance in epithelial cancers will be discussed.

## Introduction

Broadly speaking, epithelia are organized into sheets, tubes, or glandular structures, and perform complex tasks of transporting ions, organic molecules, and water for which specific ion channels/transporters are required. The majority of cancers are of epithelial origin, and the altered ion channel/transporter expression, which is emerging as one of the hallmarks of cancer in general (Prevarskaya et al., 2010; Lehen'kyi et al., 2011), is also a marked characteristic of epithelial cancers. In this review we will first outline the ion transport mechanisms operating in epithelia under physiological conditions of ion/fluid transport and cell volume regulation. Next, we will review and critically discuss how dys-regulation of cell volume or given ion transporters can lead to loss of epithelial architecture, altered cell survival, tumor progression, and drug resistance. The focus will be on cancers of secretory epithelia, primarily pancreatic ductal adenocarcinoma (PDAC) and mammary cancer.

## Physiology of epithelial transport and role of cell volume

Animal cells are subjected to transmembrane osmotic gradients in a number of physiologically relevant conditions, including: (i) ion/nutrient transport followed by osmotically obliged water movement; (ii) metabolic activity generating or requiring osmotically active substances; or (iii) altered extracellular osmolarity of the environment [see Hoffmann et al. (2009)]. Epithelial cells are of special interest because they carry out net transport of electrolytes, nutrients, and water in the secretory or reabsorptive direction, conditions in which cell volume regulation is a particular challenge. A question that has raised substantial interest in the field is how well cell volume regulation is achieved under these conditions, and to what extent cell volume changes contribute to the regulation of secretion/absorption. Furthermore, little is known about what happens to cell volume regulation if the normal vectorial epithelial transport is prevented or dys-regulated. It is well documented that several pathophysiological conditions, including altered Na^+^/K^+^ balance and acid/base disturbances caused by renal disease, or cardiac or brain ischemia, are associated with dys-regulation of cell volume regulatory transporters, and that the associated cell volume disturbance contributes importantly to the pathology of these conditions (for reviews, see Lang, 2007; Hoffmann et al., 2009; Pedersen et al., 2011).

In *absorptive* epithelia such as the renal tubules, small intestine, gallbladder, and skin, the most common mechanism of transepithelial transport involves luminal channels and transporters that utilize the plasma membrane Na^+^ gradient for salt and nutrient transport, which would tend to swell the cells. Isosmotic transport and recovery of cell volume under these conditions is likely achieved through activation of basolateral stretch-activated K^+^ channels, volume regulated Cl^−^ channels (VRAC), and increased activity of the Na^+^/K^+^ pump, followed by exit of ions/nutrients and osmotically obliged water across the basolateral membrane (Lang et al., 1998; Vanoye and Reuss, 1999; Schultz and Dubinsky, 2001; Hoffmann et al., 2009; Bachmann et al., 2011).

**Figure 1:**
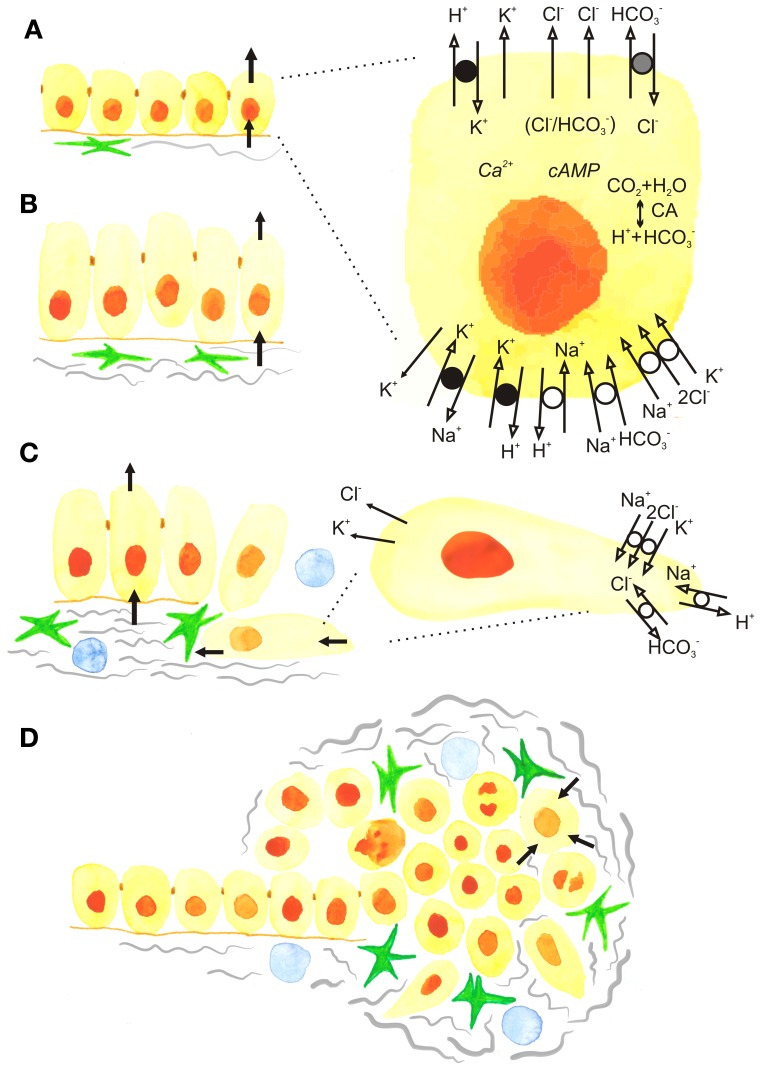
**The development of epithelial cancer and roles of ion transport and cell volume. (A)** Normal secreting epithelium showing net movements of ions and fluid across the basolateral and luminal membranes. Black arrows show the movement of ions and fluid across cell membranes. The insert shows the detailed model of a cell with basic ion channels and transporters that operate, for example, in pancreatic ducts, but are also applicable to other secreting epithelia. The luminal Cl^−^ channels include CFTR and TMEM16A/ANO1, as well as a SLC26 family Cl^−^/HCO^−^_3_ exchanger. The basolateral membrane contains the Na^+^/K^+^/2Cl^−^ transporter NKCC1, SLC7 family Na^+^-HCO^−^_3_ cotransporters (NBCs), and the Na^+^/H^+^ exchanger NHE1. The epithelium also expresses H^+^/K^+^ pumps, as well as several types of K^+^ channels such as IK-KCa3.1, BK-KCa1.1, KCNQ1 and voltage-activated K^+^ channels, some of which may be expressed on both luminal and basolateral membranes. The major Ca^2+^ and cAMP signalling pathways are not elaborated and for simplicity, Ca^2+^ -channels/transporters and aquaporins are not included. **(B)** Carcinogenesis and postulated dysregulation of cells volume and secretion. Increased activity of fibroblastic cells, such as cancer associated fibroblasts (CAFs) and pancreatic stellate cells (PSCs) (green). **(C)** The epithelial-to-mesenchymal (EMT) transition showing cells that loose apico-basal polarity and the appearance of some ion transporters from the lumimal membrane in the rear of the cells and some from the basolateral membrane in the leading edge, contributing to driving cell migration. **(D)** Progression to cancer, showing tumor with extensive fibrosis (gray), fibrogenic cells (green) and immune cells (blue). (Blood vessels are not shown). In the center of the tumor cells may die, while surrounding cells are proliferating and cell volume and corresponding ion transport is up-regulated (see the text).

Here, we will focus on *secretory* epithelia such as pancreas, salivary glands, colorectum, stomach, mammary glands, and prostate, which, as will be discussed below, might not fully regulate their cell volume during stimulated secretion. Notably, several of these epithelia are among the tissues in the body that are most commonly afflicted by cancer (Siegel et al., 2013). One of the most common mechanisms for initiating fluid secretion by agonists or hormones is opening of luminal Cl^−^ channels and luminal and basolateral K^+^ channels, and this also leads to a cell volume decrease. A number of transport mechanisms on the basolateral membrane are activated to provide ions for luminal exit and thus secretion, and this will potentially lead to regain of cell volume. Concurrently, the cells need to regulate their intracellular pH (pH_i_), and for cells exhibiting net secretion of H^+^ or HCO^−^_3_ (stomach, pancreatic ducts), this is a particular challenge. Figure 1A shows the basic model for ion transport across secretory cells such as pancreatic duct cell. As seen, this model includes a toolbox of ion channels and transporters (Novak et al., 2011; Frizzell and Hanrahan, 2012; Wilschanski and Novak, 2013), some of which are dys-regulated in cancer, as will be described below. The ion channels include: the cystic fibrosis transmembrane conductace regulator (CFTR) and Ca^2+^-activated Cl^−^ channels (ANO1/TMEM16A), intermediate and large conductance K^+^ channels (IK—KCa3.1; BK—KCa1.1), volume sensitive KCNQ1 channels, and possibly voltage-regulated channels (HERG—Kv11.1; EAG2—Kv10.2) (Hayashi et al., 2012; Wang et al., 2013). The ion transporters include Na^+^-K^+^-2Cl^−^ cotransporters (NKCC1), Na^+^/H^+^ exchangers (NHEs), Cl^−^/HCO^−^_3_ exchangers (SLC26A3,6 and SCL4A family), Na^+^-HCO^−^_3_ transporters (NBCs) and H^+^/K^+^-pumps. Another mechanism of achieving secretion, which is beyond the scope of this review, is that driven at least in part by exocytosis, such as in mammary epithelial cells secreting milk, or, for example, parietal cell secreting hydrochloric acid following exocytotic recruitment of the H^+^/K^+^ pump from tubulovesicles to the apical membrane (Forte and Zhu, 2010).

In terms of cell volume, the crucial question is how ion/fluid transport on the two opposing membranes is coordinated. The main driving force for all these secondary- or tertiary- active processes is provided by the Na^+^/K^+^-ATPase. For secretory epithelia, the classical view is that basolateral transporters are activated secondarily to ion movements across the apical membrane due to alterations in electrochemical gradients or cell volume changes. Regarding the cell volume, known shrinkage-activated proteins are NHE1, NKCC1, and some Transient receptor potential vanniloid (TRPV) channels; and swelling activated proteins are volume regulated anion channels (VRAC), KCNQ1, two-pore K^+^ channels and Ca^2+^-activated K^+^ channels (Hoffmann et al., 2009). In addition to these transporters and channels, other plasma membrane transporters are regulated by volume-sensitive signaling pathways, including intracellular messengers, phosphorylation, and complex interactions involving cytoskeletal reorganization, Ca^2+^-signaling, and signaling via integrins and receptor tyrosine kinases (RTKs). For overview of these topics the reader is referred to the recent review (Pedersen et al., 2011). Here we just point out that recently discovered cell signaling pathways involving volume- and low Cl^−^-sensitive With No Lysine kinases (WNK), acting via Ste20-like kinases, SPS-related proline/alanine-rich kinase (SPAK) and oxidative stress responsive kinase (OSR1), may be key factors in secretory epithelia as they regulate NKCC1 and other transporters (Kahle et al., 2006; Hoffmann et al., 2009; McCormick and Ellison, 2011; Park et al., 2012). Similarly, autocrine and paracrine signaling via volume-sensitive ATP release and purinergic receptors may be important regulators of key short- and long-term cell volume and ion transport in epithelia and tumor models (Hug et al., 1994; Pedersen et al., 1999; Sørensen and Novak, 2001; Koltsova et al., 2011; see Novak, 2011). A number of ATP release mechanism have been proposed, including ion channels and transporters, and they utilize favorable electrochemical gradient (see Novak, 2011).

Nevertheless, in the acute/secretory state, the cell volume of many native epithelial cells recovers only partially or does not recover until the stimulus is withdrawn (Manabe et al., 2004; Bachmann et al., 2007). For example, some secretory cells shrink by more than 20% during stimulation and remain shrunken until the stimulus is withdrawn (Dissing et al., 1990; Foskett, 1990; Nakahari et al., 1990, 1991; Lee and Foskett, 2010) (Figure 1A). The chronic events of altered volume regulation and/or ion transporter expression might lead to pathological developments associated with cancer.

## Loss of epithelial polarity—implications for ion transport

The polarized organization of ion transport proteins is essential for the normal function of epithelia, and appears to involve the interplay between the targeted delivery of transporters, restriction by cell-cell junctions, and the fact that the transporters reside in large protein-protein complexes linking them to the actin- and spectrin-based cytoskeleton (Nelson, 2009). During early stages of cancer development, the epithelial layer becomes disorganized, loses its cell-cell adhesions, and undergoes a dramatic change from apical-basal polarity to a mesenchymal cell type organization with a front-rear polarity (Figures 1B,C). This process is known as epithelial-to-mesenchymal transition (EMT), and has been well studied both for breast and pancreatic adenocarcinomas (Foroni et al., 2012; Rhim et al., 2012). Although the signaling mechanisms involved in EMT are far from fully elucidated and are partially context- and cell-type dependent, several central themes have been established. Upstream EMT features include up-regulation of transcription factors such as Slug, Snail, and Twist. Markers of the full-blown EMT include up-regulation of α-smooth muscle actin (α-SMA), vimentin, and fibronectin, and down-regulation of epithelial markers such as E-cadherin, cytokeratins, and the tight junction protein ZO-1 (Kalluri and Weinberg, 2009; Nelson, 2009; De Craene and Berx, 2013). Notably, although a number of factors involved in polarity switching are described (Nelson, 2009; Godde et al., 2010), essentially nothing is known regarding the roles and regulation of polarized transport proteins during EMT. Thus, it is an open question how the tightly compartmentalized localization of transport proteins gets “reinstructed” upon transition from apical-basal to a front-rear polarity (Figure 1C). The net result, however, is that at least some apical ion channels and transporters relocalize to the rear end, while several that are basolaterally located in epithelia move to the leading edge of the cell (compare Figures 1A,C). This specific reorganization of ion channels and transporters contributes importantly to cell migration (Schwab et al., 2012). Given the known roles of many of these transport proteins in cytoskeletal organization, signaling, and motility, we speculate that contributions to EMT might be added to the list of roles for dys-regulation of transport proteins in epithelial cancers.

### The tumor microenvironment (TME)

Tumors are highly complex tissues in which the cancer cells themselves are often the minority and co-exist with numerous other cell types in a physical/chemical microenvironment which differs dramatically from that of the normal tissue (Figure 1D). The tumor microenvironment (TME) undergoes extensive reciprocal interactions with the cancer cells and provides oncogenic signals that exacerbate cancer progression. The detailed properties of the TME have been excellently reviewed elsewhere (Mueller and Fusenig, 2004; Kalluri and Zeisberg, 2006; Pandol et al., 2009; Hanahan and Weinberg, 2011; Feig et al., 2012; Hanahan and Coussens, 2012). In the following, we set the stage for discussing the interrelationship of the TME with dys-regulated ion transport, focusing on PDAC and mammary adenocarcinoma.

### The cellular component of the TME

The predominant stromal cell type in many carcinomas, including breast cancers, is *cancer associated fibroblasts (CAFs)* (Kalluri and Zeisberg, 2006; Hanahan and Coussens, 2012). CAFs secrete extracellular matrix (ECM) components and matrix-degrading enzymes, and, being contractile, mechanically pull at the ECM, increasing its stiffness (Kalluri and Zeisberg, 2006; Hanahan and Coussens, 2012). CAFs also secrete numerous growth factors, cytokines and vascular endothelial growth factor (VEGF), stimulating tumor growth and, in general, angiogenesis (Kalluri and Zeisberg, 2006; Hanahan and Coussens, 2012), though paradoxically solid tumors show poor vascularization (see below). In PDAC, *pancreatic stellate cells* (*PSCs*) play a role similar to that of CAFs in breast cancer (Pandol et al., 2009; Feig et al., 2012), although CAFs *per se* are also present in PDAC (Scarlett, 2013). Quiescent PSCs are present in low numbers in the normal exocrine pancreas. PSCs become activated by exposure to factors secreted by the cancer cells, rendering them myofibroblast-like, highly proliferative, and motile (Pandol et al., 2009; Feig et al., 2012; Li et al., 2012). Excessive ECM deposition by PSCs is the main source of the marked desmoplasia in PDAC (Figure 1D). The PSCs also secrete growth factors, cytokines and chemokines, stimulating immune cell infiltration, angiogenesis, and cancer cell proliferation and motility (Pandol et al., 2009; Feig et al., 2012; Li et al., 2012). Infiltrating immune cells are of major importance in both mammary and pancreatic adenocarcinomas (Clark et al., 2007). Recruited *tumor-associated macrophages* release growth factors, chemokines, cytokines, and matrix-degrading enzymes, stimulating angiogenesis, cancer cell growth and invasiveness and further recruitment of pro-tumorigenic immune cells, while blocking activation of anti-tumorigenic T cells (Kalluri and Zeisberg, 2006; Pandol et al., 2009; Hanahan and Weinberg, 2011; Kees and Egeblad, 2011). Other central cellular stromal components are *endothelial cells* and *pericytes* (smooth-muscle-derived cells surrounding the endothelium). Finally, *cancer stem cells* or tumor-initiating cells have been found in the TME in both mammary and pancreatic cancer (Hermann et al., 2007; Iqbal et al., 2013).

### Chemical/physical properties of the TME

In addition to the wealth of cell types and secreted signaling factors mentioned above that sets the TME apart from the normal tissue, the TME also differs markedly from the normal tissue in its physical/chemical properties (see Harris, 2002; Heldin et al., 2004; Vaupel, 2004; Egeblad et al., 2010; Provenzano and Hingorani, 2013). Similar to the cellular component, the physical/chemical microenvironment exhibits distinct spatial heterogeneity throughout the tumor and develops dynamically as the cancer progresses. Because of the generally insufficient or collapsed tumor vasculature in many solid tumors, many areas of the TME are hypoxic or even anoxic (Harris, 2002). This has been shown directly for breast cancer (Vaupel, 2004), whereas evidence is more sparse for PDAC (see Feig et al., 2012). In conjunction with cancer-associated metabolic changes and high demand for energy and building blocks for anabolic reactions, this results in glucose deprivation, elevated lactate levels, and acidic extracellular pH (pH_e_) (Heldin et al., 2004; Vaupel, 2004). Another consequence of the inefficient tumor vasculature and lymph outflow is elevated interstitial fluid pressure. A third physical characteristic of mammary and especially pancreatic cancers is that of desmoplasia—excessive accumulation and crosslinking of fibrillar collagens. This stiffens the ECM, in turn favoring cancer progression through effects on cell motility, differentiation, proliferation, and treatment response (Egeblad et al., 2010). In PDAC tumors, which are highly fibrotic and hypovascular, it is difficult for therapeutic agents to reach the tumor cells (Feig et al., 2012; Provenzano and Hingorani, 2013). Recent studies show that enzymatic targeting of stroma, ablation of the physical barrier improves vasculature and promotes drug delivery (Provenzano et al., 2012).

It seems likely that, in addition to selecting for hypoxia resistance and increased acid extrusion capacity (section Functional interactions between the TME and ion transport dys-regulation), the physically restricted TME with elevated interstitial pressure will tend to select for increased cell volume regulatory capacity due to the increased osmotic stress exposure. However, to our knowledge, this has never been directly studied. In addition, one might expect that physical constraints, hypoxia and necrosis will influence the concentration profiles of extracellular nucleosides/-tides within the tumor, in turn affecting a spectrum of tumor resident cells via purinergic signalling (Di Virgilio, 2012) (Figure 1D).

### Functional interactions between the TME and ion transport dys-regulation

**Figure 2:**
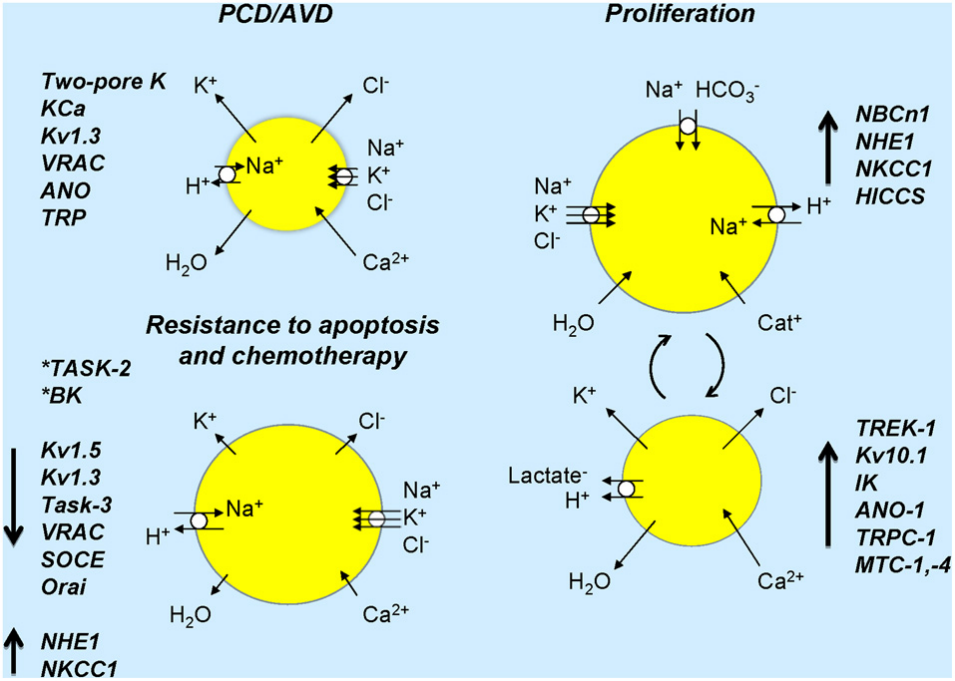
**Ion channels and transporters and cell volume changes associated in normal and cancer cells**. Cell sizes refer to expected cell volume changes and lengths of arrows on cells indicates up- or down-regulation or ion transporters/channels. Resistance to apoptosis is associated with down-regulation of several channels and inhibition of some channels (asterisks) induces resistance to apoptosis. In proliferation, several transporters and channels are up-regulated and over-expressed in cancer (see text). The right part of the figure shows ion transporters and channels that would lead to cell volume increase and those in the lower part indicate those that would lead to cell volume decrease. Large arrows next to named ion channels/transporters indicate their up- or down-regulation in cancer. *Chronic activation* of ion transport may lead to cell death. *Dynamic activation* or suppression of ion transport/cell volume with specific signals, in time or in given cells may lead to cancer development and progression.

While this has still been relatively little studied, it is clear that dys-regulation of ion transport in cancer is involved in important functional interactions with the TME. Firstly, the metabolic switch induced (in part) by hypoxia increases acid production in the cancer cells. This, in conjunction with hypoxia-induced elevation of hypoxia-inducible factor-1α (HIF1α) levels increases the expression and/or activity of acid-extruding ion transport proteins and carbonic anhydrases (CAs). In breast cancer, these include the Na^+^/H^+^ exchanger NHE1, the Na^+^-HCO^−^_3_ cotransporter NBCn1, monocarboxylate transporters MCT1 and MCT4, and CAIX (Bartosova et al., 2002; Lauritzen et al., 2010, 2012; Pinheiro et al., 2010; Boedtkjer et al., 2013; see Cardone et al., 2005) (Figure 2). In PDAC, evidence is much sparser, although neurotensin-induced NHE1 activation in PDAC cell lines is reported (Olszewski et al., 2010). Cytokines and growth factors secreted by the cancer cells and stromal cells likely also contribute to the up-regulation of ion transport. For instance, ErbB2 signaling increases NBCn1 expression and post-translationally activates NHE1 by phosphorylation in its C-terminal cytoplasmic domain (Lauritzen et al., 2010, 2012). In turn, ion transporters play major roles in creating the TME. Increased acid extrusion from the cancer cells can cause extracellular pH (pH_e_) to become as low as 6.0 in some tumor regions (Vaupel, 2004). This favors further cancer development, e.g., through facilitating ECM degradation and cell motility, resistance to chemotherapy, and compromised anti-tumor function of cytotoxic T-cells and natural killer cells (Ward et al., 2013), while their role in maintaining pH_i_ at or above the normal pH 7.0–7.4 favors metabolic, migratory, and proliferative activity and counteracts apoptotic death (Parks et al., 2011; Webb et al., 2011; Boedtkjer et al., 2012). Finally, it has been suggested that NHE1 may directly regulate ECM deposition by fibroblasts (Karydis et al., 2009).

## Roles of cell volume regulation in cell proliferation and programmed cell death (PCD)

Importantly, cells do not have one preferred volume. Rather, the volume *set point* depends on the functional state of the cell and changes in cell volume serve as key physiological signals initiating downstream responses, such as transepithelial transport (see above), proliferation, migration and cell death (Figure 2) (see Hoffmann et al., 2009). Consequently, dysfunction of volume-sensitive membrane transport proteins is associated with pathophysiological conditions related to control of these processes, including cancer.

### Cell proliferation

Cell volume is a major factor in the regulation of cell cycle progression, with cell proliferation generally being inhibited by cell shrinkage and stimulated by cell swelling, respectively (Anbari and Schultz, 1993; Dubois and Rouzaire-Dubois, 2004; Rouzaire-Dubois et al., 2005). Cell cycle progression depends on an increase in cell volume, and the capacity for regulatory volume decrease (RVD) changes during the cell cycle (see e.g., Hoffmann et al., 2009). Accordingly, cell volume was found to be greatest in the M phase and smallest in the G1 phase in CNE-2Z cells and to increase in parallel to the G1-S transition in fibroblasts (see Hoffmann et al., 2009) In Ehrlich Lettre ascites carcinoma (ELA) cells, significant water uptake and cell swelling occur in S phase (Klausen et al., 2010). The direct effects of changes in cell volume on the cell cycle control are still not clear, but it seems that RTKs and mitogen-activated protein kinases (MAPKs) play important roles. Accordingly, cell swelling induced by hyposmotic stress in general stimulates extracellular signal regulated kinase (ERK1/2), a major player in control of cell cycle progression (see e.g., Meloche and Pouyssegur, 2007; Hoffmann et al., 2009) and multiple Src family kinases are activated in response to cell swelling (Cohen, 2005). An interesting example, somewhat in contrast to the general picture given above, is described in glioma cells, where a marked premitotic cell shrinkage is necessary for the following cell division (Habela and Sontheimer, 2007).

Several types of ion channels have been implicated in the dys-regulated control of cell cycle progression in cancer (Figure 2). *TRP channels*. The resting level of [Ca^2+^]_i_ varies through the cell cycle (Schreiber, 2005). Thus, transient changes in [Ca^2+^]_i_ occur at the exit from quiescence in early G1, at the G1/S phase transition and at the exit from M phase (Munaron, 2002; Munaron et al., 2004). In some cell types, TRPC1 is proposed to be involved in Ca^2+^ influx, RVD and cell cycle progression (Golovina et al., 2001; Salido et al., 2011; Madsen et al., 2012). A variety of *K*^+^ *channels* have been implicated in the regulation of proliferation (Takahashi et al., 1993; Pei et al., 2003; Wang, 2004; Voloshyna et al., 2008) and cell cycle progression (Wang et al., 1998; Felipe et al., 2006). Accordingly, epithelial carcinomas often show high K^+^ channel activity (Patel and Lazdunski, 2004; Wang, 2004; Felipe et al., 2006). Thus increased TREK-1 channel expression is associated with abnormal cell proliferation in prostate cancer cell lines and TREK-1 may be a novel molecular target in prostate cancer (Voloshyna et al., 2008). The K_v_10.1 (KCNH1) channel, which is widely studied in cancer, is important for cell cycle progression and is regulated through the cell cycle (Pardo et al., 2012). Thus, developing specific blockers for these channels in the treatment of cancer is a promising field (Felipe et al., 2006; Li and Xiong, 2011; Pardo et al., 2012). In PDAC, in addition to K_v_10.1 (Gomez-Varela et al., 2007), expression of IK (KCa3.1) is up-regulated in cancer tissue and some PDAC cell lines in which it contributes to stimulation of cell proliferation (Jager et al., 2004). *Cl*^−^ *channels* are also involved in control of cell proliferation, and Cl^−^ channel blockers inhibit cell proliferation (Voets et al., 1995; Pappas and Ritchie, 1998; Rouzaire-Dubois et al., 2000; Shen et al., 2000; Wondergem et al., 2001; Chen et al., 2007; Klausen et al., 2010) Several studies have found that VRAC currents differ in magnitude during the cell cycle (Shen et al., 2000; Doroshenko et al., 2001; Klausen et al., 2007, 2010). In nasopharyngeal carcinoma cells, VRAC activity was found to be central in control of passage through the G1 restriction point (Chen et al., 2007). The Ca^2+^-activated Cl^−^ channel TMEM16A (ANO-1) is overexpressed in many carcinomas, including human prostate carcinoma (Liu et al., 2012) and head and neck squamous cell carcinomas, where it induces stimulation of ERK1/2 and contributes to cell proliferation (Duvvuri et al., 2012). In mammary cancer, where TMEM16A (ANO-1) is also over-expressed and supports proliferation, it is linked to EGF receptor and calmodulin-dependent kinase II signaling (Britschgi et al., 2013). Thus, specific blockers of Cl^−^ channels are also a potentially interesting field in the treatment of cancer (Duvvuri et al., 2012; Mazzone et al., 2012). Also several volume-regulatory transporters, including NHE1 (Putney and Barber, 2003) and NKCC1 (Panet et al., 2000) have been shown to exhibit cell-cycle dependent regulation and/or roles in regulation of cell proliferation, although the specific mechanisms are not fully elucidated and for NHE1 likely include effects both on pH_i_ and cell volume.

In conclusion, ion channels and transporters have been implicated in the control of cell cycle checkpoints in normal as well as cancer cells, and specific types of ion channels seem to play an important role in tumor cell proliferation. However, a comprehensive mechanistic picture of the functional relation between ion channels and cell proliferation is yet not available (Becchetti, 2011).

### Programmed cell death (PCD)

A hallmark of PCD (or its more restrictive term, apoptosis) is a marked cell shrinkage (Kerr et al., 1972), which is entitled *Apoptotic volume decrease*, or AVD (Maeno et al., 2000) (Figure 2). AVD is an early event required for triggering of full-blown apoptosis (Maeno et al., 2000; Poulsen et al., 2010), and there is strong evidence that preventing cell volume regulation after shrinkage is associated with induction of apoptosis (Lang and Hoffmann, 2012). AVD results from a loss of KCl via K^+^ and Cl^−^ channels, and concomitant loss of water (Bortner and Cidlowski, 1998; Okada and Maeno, 2001; Okada et al., 2001; Okada, 2004; Lang et al., 2007; Poulsen et al., 2010). Apoptosis thus depends on K^+^, Cl^−^ and Ca^2+^ (to activate Ca^2+^ activated K^+^ and Cl^−^ channels) channels, such as, e.g., various voltage-dependent K^+^ channels, two-pore K^+^ channels, Ca^2+^ activated K^+^-channels, VRAC, some Ca^2+^ -activated Cl^−^ channels of the ANO family and some Ca^2+^ permeable TRP channels (see Lehen'kyi et al., 2011; Lang and Hoffmann, 2012). Enhanced expression of these ion channels in cancer cells will, as described above, typically stimulate proliferation and migration, but it will in general also be expected to be pro-apoptotic. It seems to be a paradox that cancer cells manage to up-regulate channels mainly involved in proliferation and migration, while at the same time avoiding the expected pro-apoptotic effect of these channels. We favor the interpretation that proliferation /cell cycle progression is dependent on specific windows of temporal-/spatial-/signal-specific modulation of Cl^−^ and K^+^-channel activity, whereas apoptosis may be the result of a longer-term activation of Cl^−^ and K^+^-channels (Figure 2). However, elucidation of this important question will require complete characterization of the cell-cycle dependent expression- and activity pattern of the specific channels involved and mapping of their precise subcellular localization.

Proapoptotic effects of enhanced K^+^ channel expression include: (i) hyperpolarization and associated Ca^2+^ overload; (ii) AVD; and (iii) increased proteolytic cleavage of pro-caspase 3 secondary to the decrease in intracellular K^+^ (Lehen'kyi et al., 2011). The proapoptotic effect of VRAC expression is predominantly on AVD (see e.g., Poulsen et al., 2010). The TRP channels are particularly involved in the control of Ca^2+^ influx participating in the PCD process (Lehen'kyi et al., 2011). Collectively, these findings strongly indicate that ion channel dys-regulation can underlie cancer cell resistance to apoptosis (see below). This is also the case for several ion transporters. Thus, during AVD, cells lose the capacity for counteracting cell shrinkage by triggering a regulatory volume increase (RVI) response (Maeno et al., 2006), which would be normally operating in a healthy cell. In fact, in HeLa cells undergoing apoptosis, the RVI mechanism seems to be weakened (Numata et al., 2008). The transporters involved in RVI thus tend to counteracts apoptosis. As the most important transport systems in RVI are NKCC1, NHE1, the Na^+^/K^+^ ATPase, and in some cells also ENaC type cation channels (Hoffmann et al., 2009), it seems likely that increased expression or function of these in epithelial cancer would render tumor cells resistant to apoptosis, and in fact, this has been demonstrated in several types of cancers (see below).

## Ion transport and drug resistance in cancer

### Multi drug resistance (MDR)

Chemotherapy resistance—cell-intrinsic or acquired—underlies the failure of most cancer treatments. Many factors are involved in resistance of cancer cells, such as decreased drug uptake, increased drug efflux, detoxification, increased DNA repair, and dys-regulation of apoptotic signaling (Krishna and Mayer, 2000; Stavrovskaya, 2000; Lothstein et al., 2001; Giacomini et al., 2010). One of the most important contributions to drug resistance in solid tumors such as PDAC is a failure to deliver drugs due to poor vascularization of the tumor and impermeability exhibited by dense desmoplasia (see section Chemical/physical properties of the TME for details). The current strategy is to overcome both physical barriers with multi-drug therapy approach (e.g., Provenzano et al., 2012).

ATP-binding cassette (ABC) drug efflux pumps are widely studied in the context of chemotherapy resistance (see e.g., Litman et al., 2001) and will not be discussed here. As described above [sections Loss of epithelial polarity—implications for ion transport and Roles of cell volume regulation in cell proliferation and programmed cell death (PCD)], ion transporters play major roles in shaping the TME, which is, in turn, very important for drug delivery/chemotherapy resistance. The other major contribution of ion transporters in drug resistance in cancer is their role in the resistance to apoptosis, which is one of the major reasons for chemotherapy cross-resistance.

### Resistance to apoptosis

Resistance to apoptosis can develop when the AVD is prevented. This can be mediated by down-regulation of the K^+^ and/or Cl^−^ channels responsible for AVD, as well as of Ca^2+^ channels involved in Ca^2+^ influx and hence modulation of Ca^2+^ sensitive apoptotic steps. Alternatively, resistant cell can develop an enhanced RVI response, which, as described above, counteracts AVD, by up-regulation of NHE1, NKCC1, or hypertonically induced cation channels (HICCS) (Figure 2). Accordingly, it was demonstrated that Chinese hamster ovary cells, which do not perform RVI because they lack of NHE1, are more prone to apoptosis compared to cells expressing NHE1 (Rotin and Grinstein, 1989). Moreover, in HeLa cells HICCS rescue cells from staurosporine-elicited apoptosis (Numata et al., 2008). These studies underscore the critical role of volume regulation mechanisms in apoptotic resistance. Finally, although a detailed account of the roles of intracellular channels and transporters in PCD resistance is beyond the scope of this review, it may be noted that the mitochondrial voltage-dependent anion channel, VDAC-1, has been identified as a protein associated with resistance to cisplatin chemotherapy (Tajeddine et al., 2008) and has, although this remains controversial, been suggested to be part of the mitochondrial permeability transition pore, mPTP (see Javadov et al., 2011).

### The role of ion channels in chemotherapy resistance

Ion movements are important in the regulation of apoptosis, but exactly how they are involved in the development of chemotherapy resistance is not always clear; in Figure 2 and text below we summarize some molecular candidates. *Decreased K*^+^ *permeability* seems to be important cause of cancer cell resistance to apoptosis (Prevarskaya et al., 2010). For example, in PDAC, expression of Kv1.3 is down-regulated, presumably due to aberrant methylation of the Kv1.3 gene promoter, and it is postulated that this may render cells resistant to apoptosis (Brevet et al., 2009). Furthermore, the K^+^ ionophore amphotericin B counteracts cisplatin resistance in cancer cell lines (Morikage et al., 1993; Beketic-Oreskovic and Osmak, 1995) by introduction of a high K^+^ permeability, and Amphotericin B in conjunction with the NKCC blocker bumetanide was shown to augment cisplatin-induced caspase 3 activation (Marklund et al., 2000, 2001, 2004). The TASK-2 K^+^ channel blocker clofilium prevents AVD and abrogates cisplatin-induced caspase 3 activity in a cell line derived from mammary gland adenocarcinomas, Ehrlich ascites tumour cells (EATCs) (Poulsen et al., 2010). Targeting BK (KCa1.1) channels with teraethylammonium or iberiotoxin similarly attenuates cisplatin-induced apoptosis in spiral ligament fibrocytes of the cochlea (Liang et al., 2005). Several human cancers are characterized by a reduced expression of the redox-sensitive K^+^ channel Kv1.5 (Bonnet et al., 2007) and down-regulation of Kv1.5 channels in human gastric cancer cells enhances resistance to apoptosis-inducing drugs such as cisplatin (Han et al., 2007). In PDAC, Kv1.3 is down-regulated (Brevet et al., 2009). In addition, TASK-3 (Kcnk9) has been shown to have oncogenic potential in several types of human carcinomas (Pei et al., 2003). Since K^+^ channels control cell membrane potential and thus Ca^2+^ influx, the effect of down-regulating K^+^ channels on resistance to apoptosis can be also mediated by a decreased Ca^2+^ influx (see also below).

#### Decreased Cl^−^ permeability

Induction of apoptosis involves activation of VRAC in several cell types (d'Anglemont de et al., 2004, 2008; Ise et al., 2005; Poulsen et al., 2010). Moreover, some studies have shown a decrease in Cl^−^ permeability in various MDR cell models (Gollapudi et al., 1992; Lee et al., 2007; Poulsen et al., 2010; Min et al., 2011). The MDR-EATC and the KCP-4 human epidermoid cancer cells, which exhibit acquired resistance to cisplatin, both have strongly decreased VRAC activity (Lee et al., 2007; Poulsen et al., 2010). In KCP-4 cells it was further shown that restoration of the channel's functional expression leads to a decrease in the cisplatin resistance (Lee et al., 2007). Similar results were obtained in human lung adenocarcinoma cells (Min et al., 2011). In wild type EATC, cisplatin treatment induced an AVD response, whereas MDR-EATC showed almost no AVD response when treated with cisplatin (Poulsen et al., 2010). This indicates that impaired activity of VRAC channels contributes to the cisplatin resistance in MDR-EATC by preventing the necessary AVD process.

#### Ca^2+^ influx

The roles of Ca^2+^ transport in cancer and chemotherapy resistance have been excellently reviewed elsewhere (Prevarskaya et al., 2010, 2013; Dubois et al., 2013) and will only be briefly outlined here. As excessive Ca^2+^-influx contributes to PCD, conversely, preventing Ca^2+^ influx tends to help the cell to avoid PCD. In agreement with this, apoptosis-resistant prostate cancer cells have strongly reduced levels of store-operated calcium entry (SOCE) (Vanden Abeele et al., 2002; Vanoverberghe et al., 2004; Prevarskaya et al., 2013). The Orai protein is an important component of SOCE, thus down-regulation of Orai will protect the cancer cells from apoptosis. Accordingly, Orai1 was shown to contribute to the establishment of an apoptosis-resistant phenotype in prostate cancer cells (Flourakis et al., 2010).

### pH-regulatory ion transport proteins in drug resistance in cancer cells

A growing body of evidence implicates pH-regulatory ion transporters in drug resistance in cancer. The contributions of these transporters to resistance occurs at several levels. Firstly, the acidic extracellular environment in solid tumors, including the creation of a strongly acidic pericellular subdomain due to rapid H^+^ efflux (Stock et al., 2007), will, all things equal, decrease the uptake by diffusion across the plasma membrane, of chemotherapeutic drugs which are weak bases, such as doxorubicine and vinblastine, and can alter the carrier-mediated uptake of drugs via pH sensitive uptake carriers (Tredan et al., 2007). Once the drug is inside the cell, the normal-to-alkaline pH_i_, created in the tumor cytoplasm through rapid acid extrusion, impacts on the cell death machinery via multiple pathways (Pedersen, 2006). Most work in this context has been done on NHE1, inhibition or knockdown of which has been shown to enhance chemotherapeutically induced cell death in a number of cancer types (Reshkin et al., 2003; Rebillard et al., 2007; Lauritzen et al., 2010; Jin et al., 2011). Also proton pump inhibitors have been effectively used to combat chemotherapy resistance in some cancers (for a review, see De Milito and Fais, 2005), although the mechanisms are less clear, as the H^+^ V-ATPases generally predominantly localize to the endosomal/lysosomal compartments, and at least in some cancers appear to contribute little to cytosolic pH regulation (Lauritzen et al., 2010; Hulikova et al., 2013). Finally, inhibition of monocarboxylate carriers (MCTs) in cancer cells that strongly dependent on these transporters should also in principle sensitize cells to chemotherapy, however, little work has so far been done to address this directly (see Halestrap, 2013).

## Summary and perspectives

Epithelial cells are endowed with specific sets of ion channels and transporters that are organized in a polarized fashion specific for the function of the given epithelium. The molecular identities, regulation and roles of these channels and transporters in the physiology of epithelial transport and cell volume regulation are relatively well understood. Epithelial cells, no doubt due to their high proliferative rate, but perhaps also due to their continuously challenged cell volume regulation, walk a thin line between physiology and pathophysiology. We suggest, speculatively, that this may endow them with an inherently increased risk of undergoing key events contributing to development of carcinomas. It is interesting to note that in particular epithelia capable of secretion, such as prostate, mammary glands, colorectum, lung/bronchi, pancreas, stomach, and uterus seem to be frequent sites of cancer (Siegel et al., 2013). Does dys-regulation of existing ion channels/transporters, or changes in the expression of the channels lead to altered cell volume regulation and thus increased proliferation, resistance to apoptosis and chemotherapy? In this review, we have summarized existing evidence for dys-regulation of some of the important ion channels/transporters, generally from cell culture models. However, much more knowledge is needed on genuine epithelial cancer models as well as on epithelial cancers *in vivo*. The complex TME contains a number of local auto- and paracrine agents, and exhibits marked changes in pH, oxygen levels, and probably ion concentrations, compared to the normal epithelial extracellular environment. Moreover, transformed epithelial cells frequently undergo EMT, the basal membrane is degraded, and the epithelial cells come into contact with cell types they would not normally encounter. Future studies should map and functionally characterize the complete ion “transportomes” for the different cell types within the tumor, in order to uncover novel multi-therapeutic approaches to carcinoma chemotherapy.

### Conflict of interest statement

The authors declare that the research was conducted in the absence of any commercial or financial relationships that could be construed as a potential conflict of interest.

## Abbreviations

ABC: ATP-binding cassette
AVD: apoptotic volume decrease
α-SMA: α-smooth muscle actin
BK: big conductance K^+^ channel, also named KCa1.1 and maxi-K^+^
CA: carbonic anhydrase
CAFs: cancer associated fibroblasts
[Ca^2+^]_*I*_: intracellular Ca^2+^ activity
CFTR: the cystic fibrosis transmembrane conductace regulator
EATC's: Ehrlich ascites tumour cells
ECM: extracellular matrix
EGF: epidermal growth factor
ELA: Ehrlich Lettre ascites carcinoma
EMT: epithelial-to-mesenchymal transition
ERK1/2: extracellular signal regulated kinase
HICCS: hypertonicity-induced cation channels
HIF1α: hypoxia-inducible factor-1α
IK: intermediate conductance K^+^ channel, also named KCa3.1
MAPK: mitogen-activated protein kinase
MCT: monocarboxylate transporters
MDR: multi drug resistance
NBC: Na^+^-HCO^−^_3_ transporter
NHE: Na^+^/H^+^ exchanger
NKCC1: Na^+^-K^+^-2Cl^−^ cotransporter
VRAC: volume regulated Cl^−^ channel
OSR1: oxidative stress responsive kinase
pH_i_: intracellular pH
pH_e_: extracellular pH
PCD: programmed cell death
PSCs: pancreatic stellate cells
PDAC: pancreatic ductal adenocarcinoma
RVI: regulatory volume increase
RTK: receptor tyrosine kinase
RVD: regulatory volume decrease
SOCE: store-operated calcium entry
SPAK: SPS-related proline/alanine-rich kinase
TME: tumor microenvironment
VDAC-1: mitochondrial voltage-dependent anion channel
TRP: transient receptor potential channels
VEGF: vascular endothelial growth factor
WNK: with no lysine kinase
ZO-1: tight junction protein.
